# Ubiquitin-Dependent Control of Class II MHC Localization Is Dispensable for Antigen Presentation and Antibody Production

**DOI:** 10.1371/journal.pone.0018817

**Published:** 2011-04-20

**Authors:** Annette M. McGehee, Karin Strijbis, Eduardo Guillen, Thomas Eng, Oktay Kirak, Hidde L. Ploegh

**Affiliations:** Whitehead Institute for Biomedical Research, Cambridge, Massachusetts, United States of America; University of Chicago, United States of America

## Abstract

Controlled localization of class II MHC molecules is essential for proper class II MHC-restricted antigen presentation and the subsequent initiation of an adaptive immune response. Ubiquitination of class II MHC molecules on cytosolic lysine (K225) of the β-chain has been shown to affect localization of the complex. We generated mice in which the endogenous β-chain locus is replaced with a GFP tagged mutant version that lacks the cytosolic lysine residue (I-A-β-K225R-EGFP). These mice have elevated levels of class II MHC as compared to I-A-β-EGFP mice, and immature bone marrow-derived dendritic cells show redistribution of class II MHC to the cell surface. Nonetheless, in these same cells efficiency of antigen presentation is unaffected in I-A-β-K225R-EGFP mice, as assayed for presentation of ovalbumin to appropriately specific T cells. The I-A-β-K225R-EGFP animals have normal CD4 T cell populations and are capable of generating antigen-specific antibody in response to model antigens and viral infection. We therefore conclude that in our experimental system modulation of trafficking by ubiquitination of residue K225 of the β-chain is not essential for the function of class II MHC products in antigen presentation or antibody production.

## Introduction

Class II major histocompatibility complex (MHC) molecules are expressed by professional antigen presenting cells (APCs), and are necessary for the presentation of antigenic peptides to CD4 T cells and the subsequent initiation of an adaptive immune response. The timing of class II MHC-dependent antigen presentation is carefully controlled in order to achieve specificity of immune responses; the regulated trafficking of class II MHC molecules is one aspect of such control [1], [2], [3]. Ubiquitination of class II MHC molecules has recently been identified as an important mechanism that cells employ to regulate trafficking of class II MHC.

Class II MHC molecules can be ubiquitinated on a single lysine residue that is present in the cytoplasmic tail of the β-chain (I-Aβ-K225) [4], [5], [6]. Modification of the class II MHC β-chain with ubiquitin has been implicated in several steps of the trafficking pathway, including internalization, endocytic trafficking, targeting to multivesicular bodies and degradation [5], [6], [7]. Ubiquitination of class II MHC molecules occurs at the plasma membrane of immature dendritic cells resulting in recycling of class II molecules from the cell surface to internal endosomal compartments. It has been proposed that ubiquitination of class II MHC molecules is also used by dendritic cells as a mechanism to control the relocalization of class II MHC upon maturation [5]. Following engagement of Toll like receptors, and possibly other receptors as well, this ubiquitination is turned off or reversed, which results in relocalization of class II MHC from internal compartments to the cell surface of DCs [5]. This differential ubiquitination is achieved through regulation of the ubiquitin ligase responsible for the addition of ubiquitin to the class II MHC, the membrane-associated RING-CH I (MARCH-I) [7], [8], [9]. Expression of MARCH I is downregulated in human dendritic cells upon maturation [8], leading to a corresponding increase in the cell surface levels of class II MHC. In general, it appears that only a minor fraction of class II MHC products is modified by ubiquitination, with the caveat that biochemical methods are likely to underestimate the true extent of modification.

In dendritic cells, internalization of class II MHC from the cell surface is decreased when the cytoplasmic lysine of the β-chain is mutated [6]. Additionally, class II MHC molecules that lack the cytoplasmic lysine of the β-chain display a decreased localization to the internal vesicles of multivesicular bodies, suggesting that ubiquitination is important as a signal for the inclusion of class II MHC in these internal vesicles [5], [6]. Mice deficient in MARCH I show internalization of class II MHC from the cell surface of B cells, but have defects in delivery of class II MHC to acidic compartments, implying that ubiquitination may be important in the trafficking of class II MHC from early endosomes to endolysosomes [7]. Ubiquitination is probably also important for degradation of class II MHC, since cells with mutations in either MARCH I or the cytoplasmic lysine of the β-chain all showed increased levels of class II MHC, but no obvious increase in the rate of synthesis of class II MHC [5], [6], [7].

It is clear from these previous studies that the ubiquitination of class II MHC dramatically affects its trafficking. These previously published reports have not, however, addressed the physiological role of this modification in vivo in the context of the immune system. In order to address this issue we generated a targeting construct with a mutant class II MHC β-chain tagged with EGFP in which the single cytosolic lysine was replaced by an arginine (K225R). This construct was knocked-in to the endogenous I-Aβ locus, creating the I-Aβ-K225R-EGFP mouse. We show that I-Aβ-K225R-EGFP mice retain normal populations of CD4 T cells and B cells, display unaltered efficiency of antigen presentation in vitro and are capable of in vivo generation of antibody responses.

## Results

### Generation of I-A-β-K225R-EGFP mice

Ubiquitination of class II MHC regulates the localization of class II MHC molecules in both B cells and dendritic cells (DCs) [4], [6], [7]. In order to study the role of ubiquitination on the function of class II MHC molecules in vivo and in vitro, we generated a knock-in mouse model in which the endogenous class II MHC locus was replaced by a construct that encodes a mutant class II MHC β-chain that cannot be ubiquitinated (K225R).

**Figure 1:**
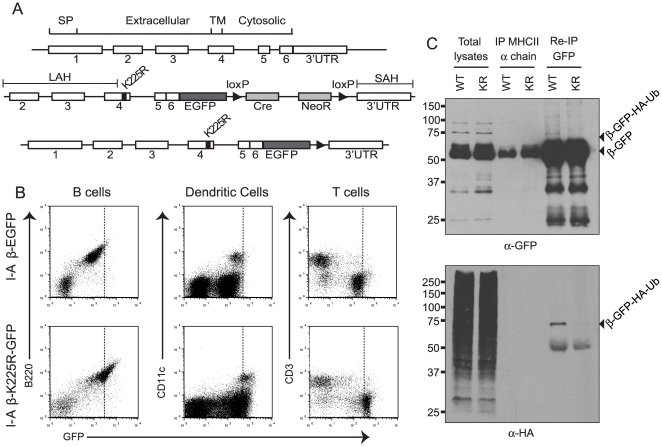
Generation of I-A-β-K225R-EGFP mice. *A*, Schematic of the approach used to target the I-A^b^-β locus. The exon structure of the I-A^b^-β locus is shown with the locations of the signal peptide (SP), extracellular domain (Extracellular), transmembrane domain (TM), and cytoplasmic tail (Cytoplasmic) indicated. The targeting construct consisted of a long arm of homology (LAH) consisting of exons 2–4, the K225R mutation introduced into exon 4, exons 5 and 6 fused to each other and to EGFP, a floxed self-excisable neomycin resistance cassette and a short arm of homology (SAH) consisting of the 3′UTR. Not drawn to scale. *B*, Splenocytes from I-A-β-EGFP or I-A-β-K225R-EGFP mice were stained with antibodies to detect B cell (B220), dendritic cell (CD11c) and T cell (CD3) populations and were analyzed by flow cytometry. Class II MHC, as detected from EGFP fluorescence, was present in B cells and dendritic cells, and class II MHC levels were increased in cells isolated from I-A-β-K225R-EGFP mice. *C*, Total splenocytes were isolated from heterozygous I-A-β-EGFP/HA-Ub and I-A-β-K225R-EGFP/HA-Ub mice and subjected to immunoprecipitation with an antibody against the MHC II α chain and subsequently to immunoprecipitation with an antibody against GFP. Total lysates were subjected to SDS-PAGE and immunoblot analysis with anti-GFP and anti-HA. All experiments were performed at least twice; representative experiments are shown.

The I-A^b^β locus was previously targeted to generate an I-Aβ-EGFP expressing mouse [10]. We chose to follow a similar strategy to generate I-Aβ-K225R-EGFP mice (Figure 1A). A construct was used in which the long arm of homology corresponds to exons 2–4 of the I-A^b^β locus, followed by fused exons 5 and 6, in frame to the coding region for EGFP. The K225R mutation in the cytoplasmic tail of class II MHC was introduced into exon 4 of this construct. The long arm of homology and a short arm of homology corresponding to the 3′ untranslated region (UTR) of I-Aβ were cloned into the pACN-TV targeting vector. pACN-TV contains a self-excisable ACN cassette that consists of a neomycin resistance gene, and a gene for Cre recombinase under the control of a sperm-specific promoter flanked by loxP sites [11]. Transcriptional induction of the ACN cassette leads to the excision of the neomycin resistance gene and Cre recombinase when the gene is transmitted through the male germline.

Correctly targeted ES cell lines, as confirmed by PCR and Southern blotting, were injected into blastocysts to generate chimeric mice, which were then bred to obtain germline transmission of the targeted I-Aβ locus. The generation of the I-Aβ-K225R-EGFP mice was confirmed by sequencing of exon 4 of I-Aβ, which showed that the I-Aβ locus in these mice does in fact harbor the K225R mutation.

The mice that received the I-Aβ-K225R-EGFP targeted allele were analyzed by flow cytometry to assess the cell specific distribution of GFP fluorescence. In splenocytes from both control I-Aβ-EGFP mice and I-Aβ-K225R-EGFP mice, all of the B220 positive B cells and CD11c positive DCs in the spleens of I-Aβ-K225R-EGFP mice were positive for GFP, while none of the CD3 positive T cells expressed GFP (Figure 1B) indicating that expression of the I-Aβ-K225R-EGFP construct was correctly restricted to the B cell and DC lineages. We consistently observed that the level of GFP fluorescence was higher in cells from I-Aβ-K225R-EGFP mice as compared to I-Aβ-EGFP controls (see below).

In order to confirm that the K225R mutation abolished ubiquitination of class II MHC molecules we crossed I-Aβ-EGFP and I-Aβ-K225R-EGFP mice with mice that express HA-tagged ubiquitin [12]. We isolated splenocytes from these I-Aβ-EGFP/HA-Ub (WT/HA-Ub) and I-Aβ-K225R-EGFP/HA-Ub (KR/HA-Ub) mice and immunoprecipitated the class II MHC complex with an antibody directed against the α chain (JV1). The eluate was used in a second immunoprecipitation with an antibody directed against GFP. Total lysates and immunoprecipitates were analyzed by SDS-PAGE and immunoblotting to detect the GFP-tagged class II MHC β chain (Figure 1C, upper panel) and HA-reactive material (Figure 1C, lower panel). We could detect a specific HA-reactive band that was present in the I-Aβ-EGFP splenocytes, but absent from the I-Aβ-K225R-EGFP splenocytes. The molecular weight of the observed HA-reactive band corresponds to the expected molecular weight of the I-Aβ-EGFP conjugated with a single ubiquitin. We conclude that the K225R mutation prevents class II MHC β chain ubiquitination in I-Aβ-K225R-EGFP mice.

### Surface and total levels of class II MHC are increased in KR mice

**Figure 2:**
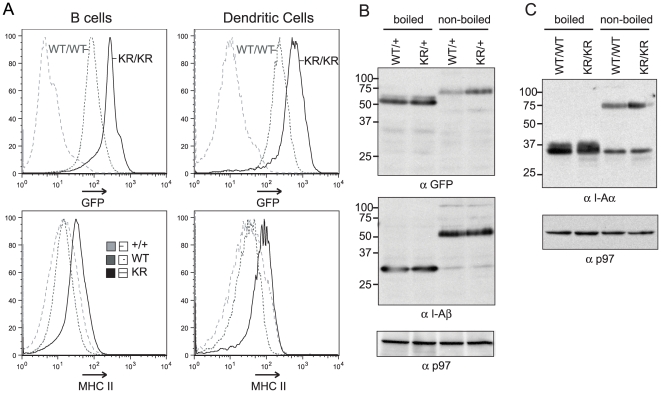
Total and cell surface levels of class II MHC are increased in I-A-β-K225R-EGFP mice. *A*, Splenocytes from B6 (+), I-A-β-EGFP (WT) and I-A-β-K225R-EGFP (KR) mice were analyzed by flow cytometry for class II levels. Total levels of class II MHC were determined from GFP fluorescence and the levels of cell surface class II MHC was assessed by staining with antibodies against I-A-β. Total and cell surface levels of class II MHC are increased in both B cells and dendritic cells of I-A-β-K225R-EGFP mice relative to I-A-β-EGFP and B6. This analysis was performed 3 times with a total of 8 mice of each genotype; representative data are shown. *B*, Splenocytes were lysed in NP40 and analyzed by SDS-PAGE. Samples were either boiled, or incubated at room temperature (non-boiled) to distinguish between the class II MHC complex and free I-Aα and I-Aβ chains. GFP-tagged I-Aβ was detected with antibodies against GFP, while the untagged I-Aβ was detected with the JV2 antibody. Levels of the mutant I-Aβ increased in I-A-β-K225R-EGFP cells while wild type I-A-β levels in the same cells remained constant. *C*, Cells were analyzed as in *B*, and immunoblotted with antibodies directed against I-Aα. Levels of I-Aα increase in I-A-β-K225R-EGFP cells showing that both components of the class II MHC molecule are affected. Immunoblotting analysis was performed at least twice; representative data are shown.

Altered ubiquitination of class II MHC molecules has been linked to changes in the levels of class II MHC that are present in the cell [5], [6], [13]. To determine the effect of blocking ubiquitination of endogenously expressed class II MHC we examined the levels of class II MHC in both B cells and DCs by flow cytometry (Figure 2A). Surface levels of class II MHC were increased in I-Aβ-K225R-EGFP mice compared to both BL6 and I-Aβ-EGFP mice. Levels of GFP fluorescence intensity of cells isolated from the I-Aβ-K225R-EGFP mice were greater than those from the I-Aβ-EGFP mice indicating that the change in surface levels reflects an overall change in levels of class II MHC, and not merely a relative relocalization of these molecules to the cell surface.

To confirm that the overall levels of class II MHC were increased in the I-Aβ-K225R-EGFP mice, we analyzed protein lysates from splenocytes by SDS-PAGE and immunoblotting (Figure 2B and 2C). Lysates were either boiled in SDS-PAGE sample buffer in order to assess single class II MHC subunits, or were not boiled in order to preserve α/β/invariant chain (Ii) and α/β/peptide complexes. In heterozygous mice that express both untargeted I-Aβ allele and a targeted I-Aβ- EGFP or I-Aβ-K225R-EGFP allele, we observed that levels of the wild type allelic product remain constant, as concluded from immunoblotting with the JV2 antibody, which recognizes the cytoplasmic tail of I-Aβ and does not recognize the EGFP tagged I-Aβ. Levels of the mutant I-Aβ-K225R-EGFP protein were elevated compared to levels of I-Aβ-EGFP, as determined from immunoblotting against the EGFP tag. Finally, I-Aα levels were also increased in I-Aβ-K225R-EGFP mice (Figure 2C). For both I-Aα and I-Aβ-EGFP greater differences were seen in the non-boiled samples, indicating that it is indeed the class II MHC complex as such that is targeted by ubiquitination, and not simply unpaired I-Aβ chains.

### KR mice show normal synthesis and maturation of class II MHC

**Figure 3:**
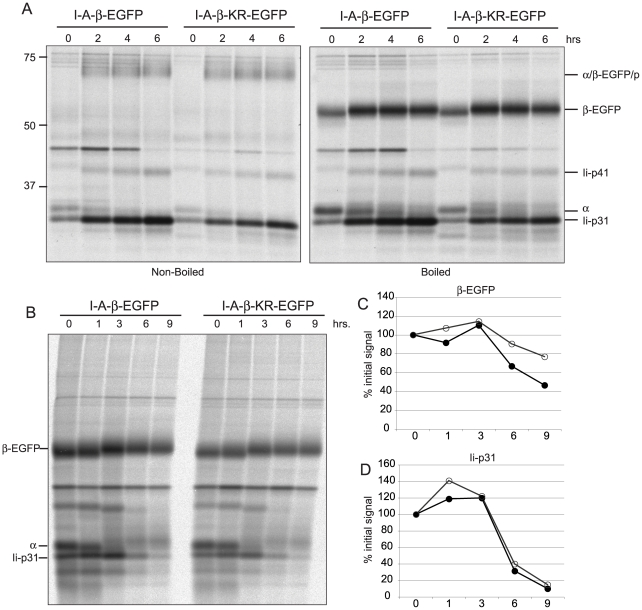
Synthesis and maturation of class II MHC is normal in I-A-β-K225R-EGFP mice. *A*, Splenocytes were pulsed with [^35^S]-cystine/methionine for 30 minutes and chased for the indicated times. Immunoprecipitations were performed with the I-Aα-specific antibody JV1, precipitates were eluted at room temperature (left panel) or boiled (right panel). *B*, Experiments were performed as in (A), immunoprecipitates were boiled to elute samples. *C and D*, Quantification of the β-EGFP and Ii-p31 data in (B). Closed circles: I-A-β-EGFP, open circles: I-A-β-K225R-EGFP. These experiments were performed three separate times; representative data is shown.

The increase in class II MHC levels in I-Aβ-K225R-EGFP mice could be caused by a change in the synthesis, maturation or degradation of class II MHC. We analyzed cells from these mice by metabolic labeling in a pulse chase experiment, followed byimmunoprecipitation (Figure 3). Rates of synthesis of class II MHC were similar in both cell types and the appearance of SDS-resistant α/β/peptide complexes occurred with comparable kinetics in the two cell types (Figure 3A), indicating that trafficking to peptide loading compartments and maturation of class II MHC molecules are not affected by the I-Aβ-K225R-EGFP mutation. Ubiquitination is therefore not required for maturation of class II MHC molecules.

Since ubiquitination has been implicated in the degradation of class II MHC molecules [5], we wanted to address whether the degradation of class II MHC molecules is altered in I-Aβ-K225R-EGFP mice. In order to address this question we looked at later time points in our metabolic labeling experiments and quantified the recovery of labeled proteins after immunoprecipitation with antibodies directed against I-Aα (Figure 3 B–D). At early time points after radiolabeling (1 and 3 hours) recovery of I-Aβ and Ii increased in both WT and KR mice, presumably due to the increased generation of α/β/Ii complexes. At later time points (6 and 9 hours) recovery of Ii decreased dramatically, suggesting that the class II MHC complexes had reached a cellular compartment in which the Ii was degraded (Figure 3B and 3D). This was true for both WT and KR mice, providing further evidence that the maturation of class II MHC molecules is normal in the I-Aβ-K225R-EGFP mice. Reduction of I-Aβ, presumably a result of class II MHC complex degradation, was seen in both WT and KR mice; however, the degradation of class II MHC tended to be greater in the WT mice (Figure 3B and 3C) in the three times that this experiment was performed, but the differences did not reach statistical significance.

### KR mutation affects the subcellular localization of class II MHC molecules

**Figure 4:**
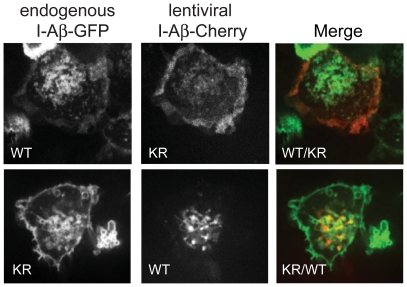
I-A-β-K225R-EGFP is redistributed to the cell surface. BMDCs from I-A-β-EGFP were transduced with I-A-β-K225R-Cherry and the localization of both molecules was visualized by confocal microscopy (top panels). This experiment was performed on four separate occasions. 39 cells expressing both proteins were visualized and 85% of these showed increased surface localization of I-A-β-K225R-Cherry compared to I-A-β-GFP. Representative pictures are shown. The bottom panels show the converse experiment in which I-A-β-K225R-EGFP BMDCs were transduced with I-A-β-Cherry. This experiment was performed twice and the results were similar to those in the converse experiment. Representative pictures are shown.

In order to determine the effects of ubiquitination on the localization of class II MHC molecules, we performed confocal microscopy on live bone marrow-derived dendritic cells (BMDCs). We took advantage of the fact that our targeted mice expressed EGFP tagged I-Aβ to allow us to visualize both wild type and K225R mutant I-Aβ in the same cell simultaneously. Dendritic cells that had been cultured for 5 days from either I-Aβ-EGFP or I-Aβ-K225R-EGFP mice were transduced with lentiviral vectors that express either I-Aβ-K225R-Cherry or I-Aβ-Cherry, respectively, to enable simultaneous imaging (Figure 4). Since the localization of class II MHC molecules changes upon dendritic cell maturation it is possible to use the localization of class II MHC molecules to assess the maturation state of dendritic cells. We therefore used the endolysosomal localization of our wild type fluorophore-tagged class II MHC molecules as a marker to identify immature dendritic cells. Localization of I-Aβ-K225R to the cell surface was dramatically increased, while localization to the endolysosomal compartments was decreased compared to the wild type construct (Figure 4). Localization of I-Aβ and I-Aβ-K225R are not affected by either the kind of fluorescent tag that is used to visualize the molecules (EGFP vs Cherry), or by the expression system (endogenous vs. lentiviral).

### Immune cell populations are not affected by KR mutation

**Figure 5:**
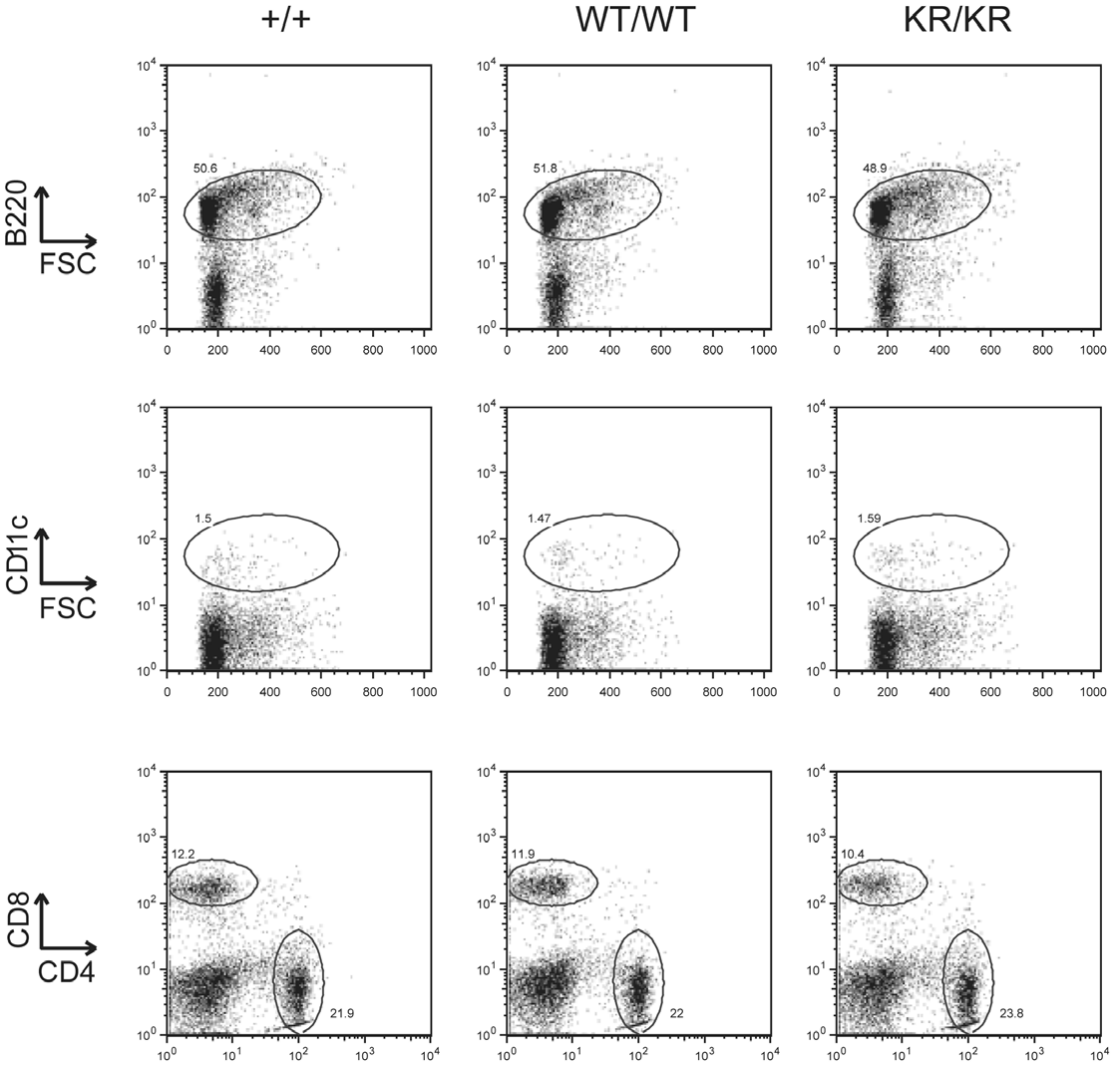
Immune cell populations are not affected in I-A-β-K225R-EGFP mice. Splenocytes from B6 (+/+), I-A-β-EGFP (WT/WT) and I-A-β-K225R-EGFP (KR/KR) mice were isolated, stained with the indicated antibodies and analyzed by flow cytometry. Shown are data from a representative experiment. This analysis was performed 3 separate times on a total of 8 mice of each genotype.

Disrupting the ubiquitination of class II MHC molecules alters both the total levels of class II MHC and their distribution. Does this change in class II MHC dynamics affect the function of class II MHC? We started to address this question by surveying lymphocyte populations in both I-Aβ-EGFP and I-Aβ-K225R-EGFP by flow cytometry. Total numbers and percentages of splenic B cells (B220), CD4 T cells, CD8 T cells and dendritic cells (CD11c) were comparable between the I-Aβ-EGFP and I-Aβ-K225R-EGFP mice (Figure 5). In addition immune cells from thymus, bone marrow, lymph nodes and the peritoneal cavity were analyzed yielding no detectable differences between the two mice (data not shown). Because class II is important for T cell development [14], we monitored populations of CD4 and CD8 negative, single positive and double positive T cells isolated from the thymus. However, no differences were detectable. In bone marrow we investigated development of B cells from the early pre pro-B cell through pre-B cell stages according to the staining scheme proposed by Hardy et al. [15]. In lymph nodes, spleen, and the peritoneal cavity we looked at total populations of B cells (B220), T cells (CD4 and CD8) and dendritic cells (CD11c). In conclusion, none of the investigated cell populations were altered in I-Aβ-K225R-EGFP mice as compared to I-Aβ-EGFP controls.

### Antigen presentation is not affected by KR mutation

**Figure 6:**
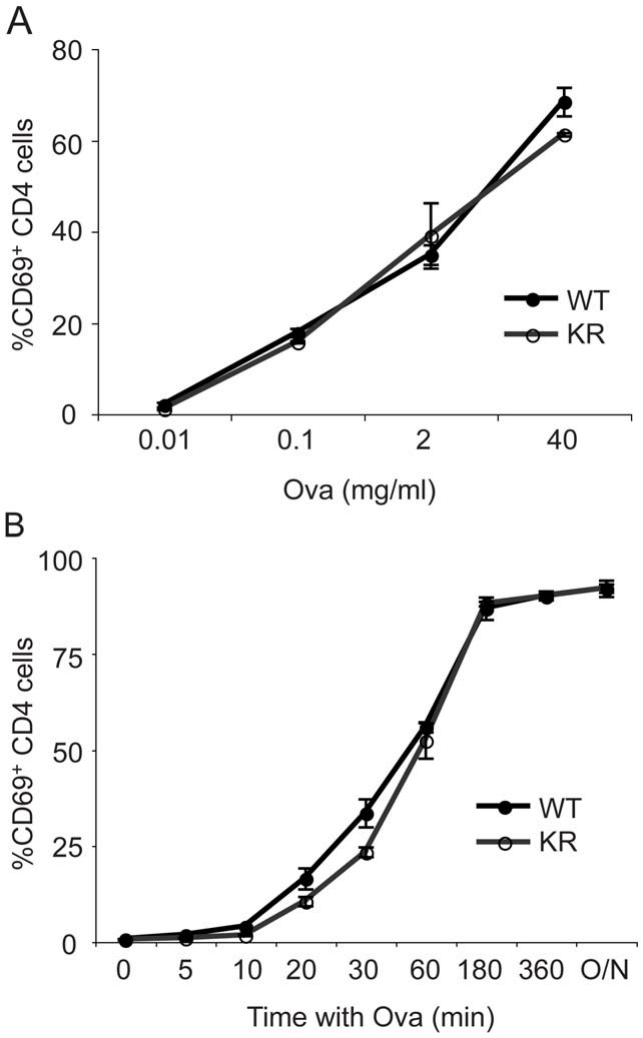
Antigen presentation efficiency is normal in I-A-β-K225R-EGFP BMDCs. *A*, BMDCs were incubated with the indicated concentrations of ovalbumin (Ova) for one hour and subsequently fixed. T cells from OTII mice were incubated with the fixed BMDCs overnight and upregulation of CD69 on T cells was assessed by flow cytometry. *B*, Assays were performed as in *A*. BMDCs were incubated with 2 µg/ml Ova for the indicated times prior to fixation. These experiments were performed twice with a total of 4 mice of each genotype; representative data are shown.

We next investigated antigen presentation by bone marrow-derived DCs from I-Aβ-K225R-EGFP mice. At day 5 the majority of BMDCs were immature as judged by low levels of cell surface CD80, and no difference was observed between the WT and KR dendritic cells (data not shown). These Day 5 BMDCs were incubated with ovalbumin (Ova) and were subsequently fixed. Fixed BMDCs were then incubated overnight with T cells isolated from OTII mice, whose T cell receptor recognizes the Ova 323–339 peptide-class II MHC complex. Activation of CD4 T cells was measured by upregulation of the cell surface marker CD69 (Figure 6). Antigen presentation by BMDCs to OTII T cells was unaffected in DCs from I-Aβ-K225R-EGFP mice. Titration of Ova added to the cells (Figure 6A), or alteration of the time allowed for processing prior to fixation (Figure 6B) did not reveal any differences in the efficiency of antigen presentation between I-Aβ-EGFP and I-Aβ-K225R-EGFP mice.

### Antibody responses are normal in I-Aβ-K225R-EGFP mice

Class II MHC molecules play an essential role in the T cell-dependent antibody response. If correct class II MHC homeostasis is important for these responses then the altered class II MHC dynamics in I-Aβ-K225R-EGFP mice could affect the ability of animals to produce antibodies.

**Figure 7:**
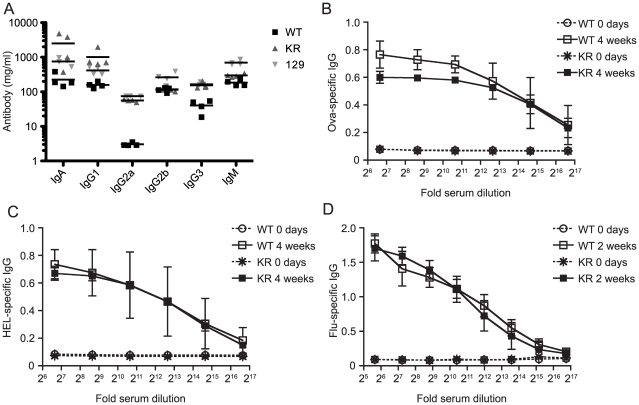
Basal and antigen specific antibody production are not altered in I-A-β-K225R-EGFP mice. *A*, Serum from peripheral blood was analyzed for immunoglobulin titers for each of the indicated isotypes by ELISA. *B and C*, Mice were immunized with 10 µg hen egg lysozyme (HEL) and 10 µg Ova on day 1 and day 21. Serum from peripheral blood was obtained on day 1 prior to immunization and from the same mice on day 28. Ova (*B*) and HEL (*C*) specific IgGs were detected by ELISA. *D*, Mice were infected with the influenza virus on day 1. Serum from peripheral blood was obtained on day 0 and on day 14. Flu specific IgGs were detected by ELISA. All experiments were performed two separate times with a total of 8 mice of each genotype; representative data are shown.

We measured the levels of IgA, IgG1, IgG2a, IgG2b, IgG3 and IgM in serum from 129, I-Aβ-EGFP, and I-Aβ-K225R-EGFP mice (Figure 7A). No differences were noted in the serum levels in any of the immunoglobulin isotypes, except for an increase in IgG2a in the I-Aβ-K225R-EGFP mice. However, since the higher IgG2a titers were also present in 129 mice, and the I-Aβ-K225R-EGFP mice are on a mixed 129/BL6 background, this difference is likely to be attributable to a difference in strain background rather than to the difference in I-Aβ.

In order to assess antigen-specific immunoglobulin production, mice were immunized with Ova and hen egg lysozyme (HEL). Serum was isolated from mice prior to immunization, again after a 4-week course of immunization, and was then analyzed for the presence of HEL- and Ova-specific IgG (Figure 7B–C). The antigen-specific IgG response varied between individual mice of each group, but we did not observe changes in the antibody response between the two groups of mice.

In order to test the ability of the I-Aβ-K225R-EGFP mice to generate an antibody response under more physiologically relevant infection conditions we assessed their ability to produce antibodies in response to infection with influenza. The influenza-specific antibody response was measured 14 days after inoculation with the influenza virus. There was no difference in the generation of influenza-specific IgG between I-Aβ-EGFP and I-Aβ-K225R-EGFP mice (Figure 7D). In conclusion, our results indicate that the I-Aβ-K225R-EGFP mice are able to produce an antigen-specific response after both immunization and viral infection that is comparable to wild type at this level of analysis.

## Discussion

Previous studies have shown that class II MHC is ubiquitinated on the β-chain, which helps control the localization and expression of class II MHC molecules [4], [5], [6]. These previously published results were obtained in cultured cells and did not address the physiological role of class II MHC ubiquitination in vivo.

In order to understand the role of class II MHC ubiquitination in the context of the immune system we generated a mouse in which the endogenous I-Aβ locus was replaced with a mutant version of I-Aβ that lacks the cytosolic lysine residue that is essential for ubiquitination (I-Aβ-K225R-EGFP). As expected, we found that the K225R mutation abolishes ubiquitination of the class II MHC β chain in splenocytes. I-Aβ-K225R-EGFP mice have increased class II MHC levels due to decreased degradation, and show an altered distribution of class II MHC between endolysosomal compartments and plasma membrane in immature bone marrow-derived dendritic cells.

Several possibilities can be envisioned for how altered trafficking dynamics might affect class II MHC function: altered dynamics of class II MHC recycling may affect the ability to present antigens to T cells, alterations in the levels and efficiency of class II MHC molecules in the thymus could lead to skewing of the overall CD4 T cell population. In the context of an immune response, the ability of animals to produce antibodies in response to antigen challenge could be altered.

However, altered localization and trafficking of class II MHC in bone marrow derived dendritic cells from I-Aβ-K225R-EGFP mice did not affect antigen presentation to T cells, notwithstanding increased levels of class II MHC at the plasma membrane. Perhaps the population of class II MHC that is ubiquitinated is intended for degradation, or destined for some other fate that is not immediately relevant for the efficiency of antigen presentation in BMDCs.

A recent report by Ohmura-Hoshino et al. [16] addressed the role of class II MHC ubiquitination in cytokine secretion and antigen presentation by dendritic cells. These authors found that dendritic cells isolated from the spleens of either MARCH I ubiquitin ligase knock out animals, or K225R knock-in animals had defects in the secretion of IL-12 and TNF-α, and in the presentation of ovalbumin to OTII T cells. Surprisingly, bone marrow derived dendritic cells from these same animals did not show any alterations in IL-12 secretion. However the ability of these BMDCs to present antigens was not addressed in the paper. We found that BMDCs are equally capable of antigen presentation regardless of whether they express wild type or K225R I-Aβ. However, the distribution of I-Aβ-EGFP in immature bone marrow derived dendritic cells is clearly affected by the K225R mutation. These results suggests that antigen presentation itself is not affected by ubiquitination of class II MHC, at least not in a way that can be read out by the assays used here.

The differences in antigen presentation between splenic and bone marrow-derived DCs observed by Ohmura-Hoshino et al. may be partially attributable to differences in DC development. In addition, MARCH I may well target substrates other than class II MHC. Using our in vitro differentiation protocol we have bypassed the effects of DC interaction with the splenic microenvironment, and thus we can argue that our system allows us to directly investigate the effect of class II MHC ubiquitination on antigen presentation, rather than on dendritic cell development.

We found that development and numbers of CD4 and CD8 T cells are normal in the I-Aβ-K225R-EGFP mice. If the dendritic cells in the thymus were defective in antigen presentation a change in the number of CD4 T cells would be expected [17], however this is not the case, indicating that differential localization of class II MHC does not measurably affect CD4 T cell selection.

Additionally, we find that antibody responses are normal in mice that cannot ubiquitinate class II MHC. Antibody generation requires efficient class II MHC-mediated antigen presentation by both B cells and dendritic cells, and thus the ability of the I-Aβ-K225R-EGFP to generate antibody responses indicates that in vivo the level of antigen presentation by class II MHC is sufficient at least to support the antibody response.

In conclusion, our experiments yielded no evidence that the alterations in class II MHC levels and their trafficking affected efficiency of antigen presentation per se, or the ability of mice to generate antibody responses. As previously published results imply a role for class II MHC ubiquitination in the regulation of dendritic cell functions in vivo [16], further studies of in vivo immune responses are necessary to elucidate the effects of altered class II MHC dynamics on the immune system.

## Materials and Methods

### Mouse Lines

C57BL/6J mice were obtained from The Jackson Laboratory. I-Aβ-EGFP mice [10] HA-Ub mice [12] and OTII mice [18] have been described. All animals were maintained according to the guidelines of the MIT Committee on Animal Care (CAC). These studies were approved by the MIT CAC (protocol #1005-070-08).

### Cell Culture

Bone marrow dendritic cells (BMDCs) were prepared by isolating cells from the femurs and tibias of mice. Cells were cultured in DMEM (Invitrogen Life Technologies) supplemented with 10% FBS, penicillin/streptomycin, 10 ng/ml recombinant mouse GM-CSF (Peprotech), and 1 ng/ml recombinant mouse IL-4. Media was partially replaced every two days. BMDCs were used for experiments after 5 days in culture.

### ES cell targeting and mouse generation

The targeting vector pACN-TV [11] was used for the construction of the I-Aβ-K225R-EGFP targeting construct. The long arm of homology consisted of a region spanning exons 2–4, exons 5–6 were fused together and also fused to EGFP, the short arm of homology consisted of the 3′ untranslated region of the class II MHC mRNA, these were the same as used previously [10]. The K225R mutation was introduced into the long arm of homology in pBluescript using the Quickchange XL kit from (Stratagene) and the primers: fwd-CCGTCACAGGAGTCAGAGAGGTGAGGAGCTC, rev-GAGCTCCTCACCTCTCTGACTCCTGTGACGG. The long arm of homology was inserted upstream of the ACN cassette, while the short arm of homology was inserted downstream of the ACN cassette. The diptheria toxin (DT) negative selection marker was cloned from this pACN-TV and a second DT gene was added into the construct after the short arm of homology so that the targeting construct was flanked by DT selection genes. Linear targeting constructs were introduced into C57BL/6J/129 F1 ES cells [19]. Positive ES cells were selected for by the presence of neomycin, and were then screened by PCR for the presence of the correctly targeted short arm of homology, the sequences of the primers used were: fwd-GATTCGCAGCGCATCGCCTTCTATC, rev-GTCAGCTGCACCACTTCTGTTAGGTCTCAC. The positive ES cell lines were further screened by southern blotting for the correctly targeted long arm of homology using the neomycin resistance gene as a template for probe generation. The generation of the I-Aβ-K225R-EGFP mice was confirmed by sequencing of exon 4 of I-Aβ using the following primers to amplify the region, and the fwd primer to sequence the region: fwd-TATACACTGGGGCCCTGGAACTTG and rev-CAGCTCCTCGCCCTTGCTCAC.

### Immunoprecipitation for ubiquitination status of class II MHC

I-Aβ-EGFP mice [10] and I-Aβ-K225R-EGFP mice were crossed with mice expressing hemagglutinin-tagged ubiquitin (HA-Ub) [12]. Total splenocytes were lysed in Nonidet P-40 buffer (25 mM Tris pH 7.4, 150 mM NaCL, 5 mM MgCl_2_, 0.5% NP40) supplemented with protease inhibitor cocktail (Roche) and 10 mM N-Ethylmaleimide (NEM). The I-Aβ complex was immunoprecipitated with JV1 antiserum (directed against the class II MHC α chain) and ProtA beads (Repligen). The protein complex was eluted off the beads by addition of a small volume NP40 buffer with 1% SDS, 20 mM DTT and10 mM NEM. The eluate was subjected to a second round of immunoprecipitation with an antibody directed against GFP (Abcam) and ProtA beads. Total lysates and immunoprecipitates were subjected to SDS-PAGE and immunoblot analysis.

### Lentivirus preparation and transduction

Virus preparation and transductions were performed as described previously [20]. Briefly, virus was produced in human embryonic kidney (HEK) cells 293T (American Type Culture Collection; CRL-11268) and viral supernatants were concentrated by centrifugation. Virus was added to BMDCs after 2 days in culture. After 12 h of infection, the medium was replaced with fresh medium. BMDCs were used at day 5.

### Confocal Microscopy

Images were acquired using a spinning disk confocal microscope and a 3-W water-cooled laser with an acoustic-optic tunable filter (Prairie Technologies). The system incorporated a Nikon TE2000-U inverted microscope using a Nikon ×100 magnification, 1.4 numerical aperture, differential interference contrast oil lens. Nikon type A immersion oil was used (Nikon). Images were acquired and processed using Metamorph software (Molecular Devices). Images were subsequently cropped in Adobe Photoshop and figures were constructed in Adobe Illustrator.

### FACS analysis

Cells were incubated with Fc block antibody (BD Pharmingen) before FACS staining. Antibodies used for FACS staining were anti B220-PerCP-Cy5.5, anti B220-APC, anti CD11c-APC, anti CD3-PE, anti CD4-APC, anti CD8-PerCP-Cy5.5, CD69-PE, anti-I-A^b^β-PE (BD Pharmingen). Data were collected on an LSR II flow cytometer (BD Biosciences) and analyzed with FlowJo software (TriStar).

### Metabolic labeling and immunoprecipitation

Pulse-chase experiments were performed as described [21]. Briefly, isolated B cells were starved in methionine- and cysteine-free media, then pulse-labeled with [^35^S]-methionine/cystine (Perkin-Elmer) for 30 minutes. After labeling, cells were incubated in chase medium containing unlabeled methionine (2.5 mM) and cysteine (0.5 mM). At the end of each chase interval, cells were lysed in NP-40 lysis buffer (50 mM Tris, pH 7.4, 0.5% NP-40, 5 mM MgCl_2_, 150 mM NaCl,) supplemented with protease inhibitor cocktail (Roche). Lysates were then analyzed by immunoprecipitation with rabbit anti-I-Aα serum (JV1) [22] and subjected to SDS-PAGE and fluorography. Quantitation was performed using a phosphorimager, and ImageJ analysis software (National Institute of Health).

### Cell lysis and immunoblotting

Cells were lysed in NP-40 lysis buffer supplemented with protease inhibitor cocktail. The protein concentrations of the supernatants were determined by BCA assay (Pierce). Samples were boiled in SDS-PAGE sample buffer (62.5 mM Tris-HCl, pH 6.8; 2%SDS; 10% glycerol; 0.1% bromophenol blue) with β-ME and separated by SDS-PAGE. Proteins were transferred to polyvinylidene difluoride membranes, blocked in 2% (wt/vol) milk in PBS, and immunoblotted with the indicated antibodies and appropriate horseradish peroxidase-conjugated secondary antibodies. Antibodies used are rabbit anti-GFP antibody (Abcam), rabbit anti-I-Aα serum (JV1), rabbit anti-I-Aβ serum (JV2) [22], anti-p97 (Fitzgerald Industries International). Following three washes in PBS-Tween 20 (0.1%), the blots were developed using Western Lighting Chemiluminescence Reagent (Perkin-Elmer).

### Antigen presentation assay

Day 5 BMDCs were incubated with ovalbumin at the concentrations and times indicated, BMDCs were subsequently fixed with 3% paraformaldehyde and then extensively washed. T cells were isolated from the lymph nodes and spleens of OT-II mice using the Pan T Cell Isolation Kit (Milteni Biotec). T cells were incubated with fixed BMDCs overnight. Cells were harvested by pipetting and were stained for flow cytometry with vβ5.1+5.2-FITC, CD4-FITC, CD69-PE (BD Pharmingen). Data was collected on FACS Calibur flow cytometer (BD Biosciences) and analyzed with FlowJo software (TriStar). Cells were gated on CD4 positive cells and analyzed for surface expression of CD69.

### Immunizations

On day 1 mice were immunized intraperitoneally with 10 µg each of Ovalbumin (Ova) and lysozyme (HEL) mixed with complete Freund's adjuvant (Sigma) and on day 21 with 10 µg each of Ova and HEL mixed with incomplete Freund's adjuvant. Sera were collected on day 0 and 28 for analysis by ELISA.

### Influenza infections

Mice were anesthetized by a single dose of 0.4–0.6 mg/g body weight of avertin (1.25% tribomoethanol, GIBCO), delivered by intraperitoneal injection. Infections were accomplished by intranasal instillation of influenza virus (Influenza A/PR8/34 or Influenza A/WSN/33) in up to 50 µl of PBS over a 10 minutes period. Weight of infected animals was monitored daily. Blood was extracted from the tail vein or by cardiac puncture after the animals were sacrificed.

### ELISAs

Plates were coated with either anti-mouse Ig antibodies (Southern Biotech), Ova, HEL or whole influenza particles. Bound antibodies were detected with HRP-conjugated isotype specific antibodies (Southern Biotech). ELISA plates were read using SpectraMax M2 microplate reader (Molecular Devices).

## References

[1] Siemasko K, Clark MR (2001). The control and facilitation of MHC class II antigen processing by the BCR.. Curr Opin Immunol.

[2] Kim YM, Pan JY, Korbel GA, Peperzak V, Boes M (2006). Monovalent ligation of the B cell receptor induces receptor activation but fails to promote antigen presentation.. Proc Natl Acad Sci U S A.

[3] Pierre P, Turley SJ, Gatti E, Hull M, Meltzer J (1997). Developmental regulation of MHC class II transport in mouse dendritic cells.. Nature.

[4] Ohmura-Hoshino M, Matsuki Y, Aoki M, Goto E, Mito M (2006). Inhibition of MHC class II expression and immune responses by c-MIR.. J Immunol.

[5] Shin JS, Ebersold M, Pypaert M, Delamarre L, Hartley A (2006). Surface expression of MHC class II in dendritic cells is controlled by regulated ubiquitination.. Nature.

[6] van Niel G, Wubbolts R, Ten Broeke T, Buschow SI, Ossendorp FA (2006). Dendritic cells regulate exposure of MHC class II at their plasma membrane by oligoubiquitination.. Immunity.

[7] Matsuki Y, Ohmura-Hoshino M, Goto E, Aoki M, Mito-Yoshida M (2007). Novel regulation of MHC class II function in B cells.. Embo J.

[8] De Gassart A, Camosseto V, Thibodeau J, Ceppi M, Catalan N (2008). MHC class II stabilization at the surface of human dendritic cells is the result of maturation-dependent MARCH I down-regulation.. Proc Natl Acad Sci U S A.

[9] Thibodeau J, Bourgeois-Daigneault MC, Huppe G, Tremblay J, Aumont A (2008). Interleukin-10-induced MARCH1 mediates intracellular sequestration of MHC class II in monocytes.. Eur J Immunol.

[10] Boes M, Cerny J, Massol R, Op den Brouw M, Kirchhausen T (2002). T-cell engagement of dendritic cells rapidly rearranges MHC class II transport.. Nature.

[11] Bunting M, Bernstein KE, Greer JM, Capecchi MR, Thomas KR (1999). Targeting genes for self-excision in the germ line.. Genes Dev.

[12] Ryu KY, Maehr R, Gilchrist CA, Long MA, Bouley DM (2007). The mouse polyubiquitin gene UbC is essential for fetal liver development, cell-cycle progression and stress tolerance.. Embo J.

[13] Young LJ, Wilson NS, Schnorrer P, Proietto A, ten Broeke T (2008). Differential MHC class II synthesis and ubiquitination confers distinct antigen-presenting properties on conventional and plasmacytoid dendritic cells.. Nat Immunol.

[14] Sebzda E, Mariathasan S, Ohteki T, Jones R, Bachmann MF (1999). Selection of the T cell repertoire.. Annu Rev Immunol.

[15] Hardy RR, Carmack CE, Shinton SA, Kemp JD, Hayakawa K (1991). Resolution and characterization of pro-B and pre-pro-B cell stages in normal mouse bone marrow.. J Exp Med.

[16] Ohmura-Hoshino M, Matsuki Y, Mito-Yoshida M, Goto E, Aoki-Kawasumi M (2009). Cutting edge: requirement of MARCH-I-mediated MHC II ubiquitination for the maintenance of conventional dendritic cells.. J Immunol.

[17] Nakagawa T, Roth W, Wong P, Nelson A, Farr A (1998). Cathepsin L: critical role in Ii degradation and CD4 T cell selection in the thymus.. Science.

[18] Barnden MJ, Allison J, Heath WR, Carbone FR (1998). Defective TCR expression in transgenic mice constructed using cDNA-based alpha- and beta-chain genes under the control of heterologous regulatory elements.. Immunol Cell Biol.

[19] Beard C, Hochedlinger K, Plath K, Wutz A, Jaenisch R (2006). Efficient method to generate single-copy transgenic mice by site-specific integration in embryonic stem cells.. Genesis.

[20] Vyas JM, Kim YM, Artavanis-Tsakonas K, Love JC, Van der Veen AG (2007). Tubulation of class II MHC compartments is microtubule dependent and involves multiple endolysosomal membrane proteins in primary dendritic cells.. J Immunol.

[21] Rehm A, Stern P, Ploegh HL, Tortorella D (2001). Signal peptide cleavage of a type I membrane protein, HCMV US11, is dependent on its membrane anchor.. Embo J.

[22] Driessen C, Bryant RA, Lennon-Dumenil AM, Villadangos JA, Bryant PW (1999). Cathepsin S controls the trafficking and maturation of MHC class II molecules in dendritic cells.. J Cell Biol.

